# Beyond genes: CRISPR/Cas9 for chromosomal engineering in polyploids

**DOI:** 10.3389/fgeed.2026.1916031

**Published:** 2026-08-14

**Authors:** Mahnoor Jamil, Rizwana Mqabool, Sultan Habibullah Khan, Wang-Qi Huang, Xiu-Hua Chen

**Affiliations:** 1 International Agriculture Research Institute, Yunnan Academy of Agricultural Sciences, Kunming, China; 2 National Engineering Research Center for Ornamental Horticulture, Yunnan Flower Breeding Key Laboratory, Flower Research Institute, Yunnan Academy of Agricultural Sciences, Kunming, Yunnan, China; 3 Center for Advanced Studies in Agriculture and Food Security, University of Agriculture, Faisalabad, Pakistan; 4 Department of Plant Breeding and Genetics, University of Agriculture, Faisalabad, Pakistan; 5 Center of Agricultural Biochemistry and Biotechnology, University of Agriculture, Faisalabad, Pakistan; 6 Yunnan Leader Bio-Technology Co., Ltd., Yanshan, Yunnan, China

**Keywords:** chromosome engineering, CRISPR/Cas9, satellite DNA, VIGE, viral vectors

## Introduction

Genome editing using CRISPR/Cas-based systems has revolutionized plant genome engineering by enabling precise and targeted modifications in plant genomes. The CRISPR/Cas systems have been in action for more than a decade, creating precise and targeted edits in the plant genome. However, these efforts have been mainly focused on gene-level modifications resulting in indels and substitutions of a few base pairs at the target sites. Recent advances in the field of genome editing have led to the development of various editing platforms that have driven unmatched progress in achieving the transition from small DNA sequence changes to large-scale DNA alterations including large segmental insertions, deletions, inversions and translocations. For instance, fusion of catalytically inactive Cas protein with recombinases or transposases facilitates the insertions of several kilobase long DNA segments (Yaşar et al., 2026). Likewise, prime editing has further expanded this scope by enabling deletion, replacement and even inversion of DNA regions spanning from few kilobases to megabases (Zhao et al., 2025; Jamil et al., 2026).

Moving beyond segmental DNA modifications to the chromosome-level engineering, a previous study has reported CRISPR/Cas9-mediated chromosome truncation, loss of chromosome and segmental duplications in tomato, but these phenomena were observed just as a result of defective repair pathway after induction of double-strand breaks by CRISPR/Cas9. This study mainly focused on understanding the repair of double-stranded breaks generated by CRISPR/Cas9 and what effects unrepaired breaks can have on the plant, providing evidence for CRISPR/Cas9-mediated chromosomal engineering in plants but these modifications were achieved at a very low frequency due to targeting of single-copy gene (Samach et al., 2023). In another study, a technique named CRISPR-Kill has been used to target functional repetitive DNA and induce cell death, blocking organogenesis in a spatio-temporal manner in *Arabidopsis thaliana.* The cell death observed in these studies is the outcome of chromosomal damage caused by CRISPR/Cas9-mediated targeting of repetitive sequences (Schindele et al., 2022; Gehrke et al., 2023). Although this study targeted repetitive sequences by CRISPR/Cas9 system for plant genome engineering at the chromosomal level, it was exploited only in model plant. There is not even a single report on CRISPR/Cas-mediated genome editing utilizing repeat sequences for targeted full chromosome engineering, in particular, removal of complete chromosome in the large and complex genomes of polyploid crops. However, a recent study has tapped this unexplored horizon and exhibited large-scale chromosomal engineering by using BSMV (Barley stripe mosaic virus) for delivery of CRISPR/Cas9 machinery in wheat (Chen et al., 2026). In this opinion, we discuss how Virus-Induced Genome Editing (VIGE) mediated chromosomal engineering may help to open unprecedent opportunities aimed at accelerating the plant breeding strategies, challenges associated with its implication and strategies to overcome these limitations.

## VIGE engineers alien and native chromosomes in wheat

Recently, Chen *et al.* have reported the CRISPR/Cas9-based VIGE as an efficient tool for higher-order chromosomal alterations that can remodel chromosomal architecture by targeting the satellite repeat regions present in the wheat genome (Chen et al., 2026). The novelty of this work lies in two major aspects; eliminating the whole chromosome from complex polyploid genome of wheat and presenting VIGE as a potential platform for chromosomal engineering in plants rather than just a gene-editing tool. The authors performed initial experiments in Cas9-expressing wheat line carrying the dispensable rye B chromosome, so that the lethal effects of genetic loss can be minimized. In this study, Barley stripe mosaic virus (BSMV) was used to deliver the gRNA targeting the E3900 satellite present on the long arm of B chromosome. Genotyping of the inoculated plants revealed that this editing resulted in terminal deletions, and 9.8% of plants have lost the sub-telomeric region distal to E3900 satellite, which was further validated by whole genome short-read sequencing (WGS) and fluorescence *in-situ* hybridization (FISH) analysis. Interestingly, one of the plants showing terminal truncation also indicated a duplication event involving the D1100 array, which could possibly be explained by the breakage-fusion-bridge cycle. After demonstration of successful terminal truncation, BSMV-mediated VIGE was employed to target the pSc200 satellite located on the short arm of rye-wheat Robertsonian translocation chromosome 1RS/1BL in Cas9-expressing wheat line. 94% of the inoculated plants exhibited reduction in pSc200 signals in FISH analysis. 50% of BSMV inoculated plants showed pSc200 deletion on at least one 1RS/1BL chromosome while 17% of plants exhibited deletion on both 1RS/1BL chromosomes. Notably, it was also found that repeat arrays adjacent to pSc200 remained unaffected but showed 50% less signals as compared to the wild-type plant. In addition to it, FISH analysis also revealed that broken DNA ends were sealed by *de-novo* telomere addition in edited plants hinting towards the chromosomal healing after CRISPR/Cas9 targeting. Furthermore, this strategy also eliminated an entire chromosome by targeting centromeric array *Bilby* on 1RS/1BL chromosome and achieved chromosomal elimination in 25% plants. Besides the removal of whole chromosome, short and long-arm monocentric isochromosomes were also observed in some edited plants, which reveals that targeting the centromeric repeats may also result in an increased number of chromosomes. Interestingly, the analysis for specificity of VIGE-mediated chromosomal alterations showed that the progeny of selfed 1RS/1BL monosomic plants had all wheat chromosomes but lacked only rye-B chromosome. After extensive study on alien chromosomes in wheat to demonstrate the feasibility of VIGE-mediated chromosomal alterations, Chen *et. al* investigated the applicability of the strategy to wheat native chromosomes. They successfully achieved chromosomal rearrangements and chromosome elimination in Cas9-overexpressing wheat line by targeting centromeric satellite Cent566, resulting in monosomic telocentric 5BS chromosome, monosomic 5BL/5BL isochromosome and monosomic 5B chromosome (Chen et al., 2026). This study is fascinating as it suggests that strategic selection of target sites for editing in plant genome may help to achieve the large-scale chromosomal engineering.

## Satellite DNA as a promising target for chromosomal engineering

**FIGURE 1:**
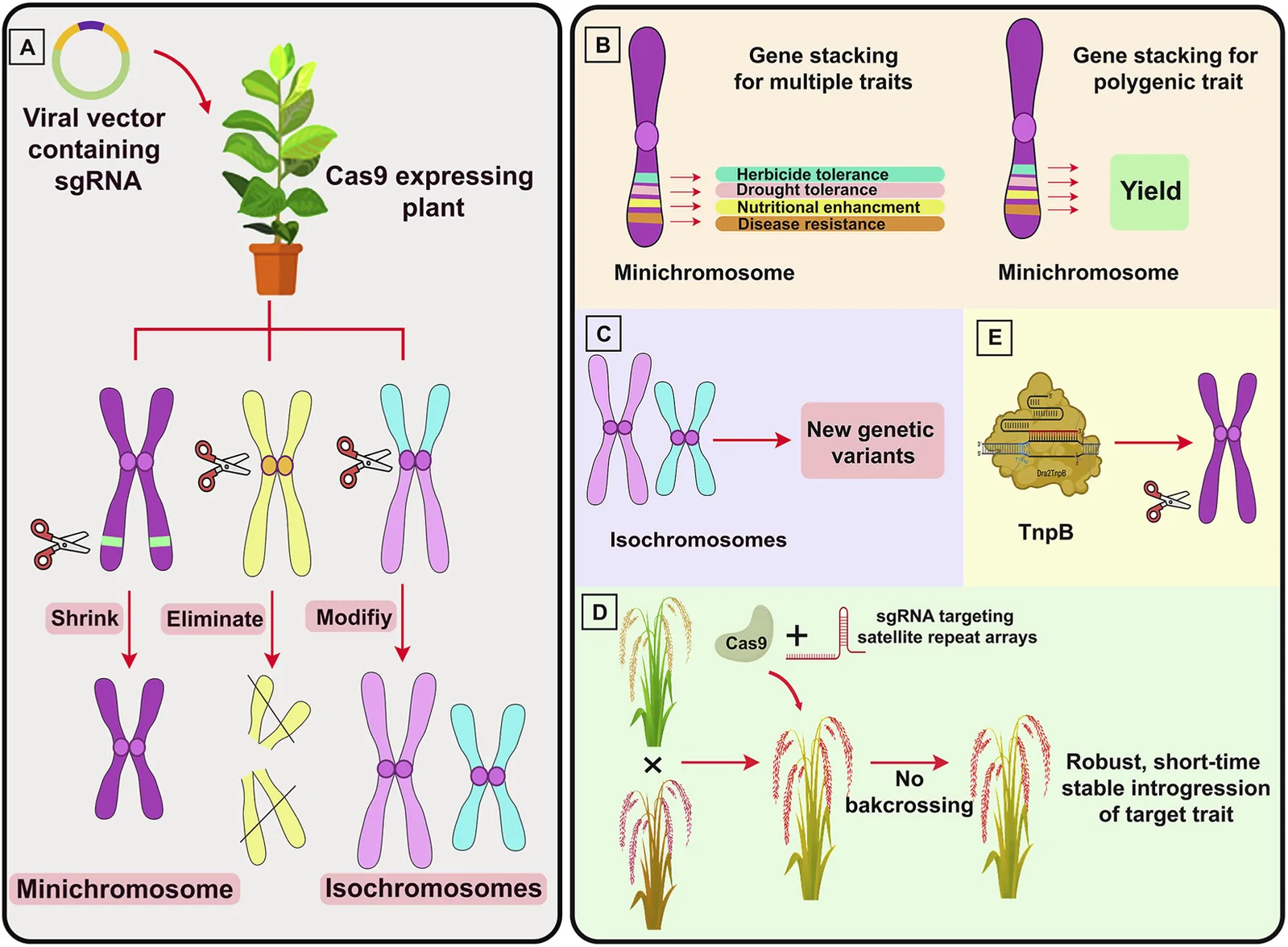
CRISPR/Cas9-mediated chromosomal engineering. **(A)** Delivery of sgRNA targeting satellite repeat arrays in the chromosomal arm removes the distal part of chromosome and results in terminal truncation generating minichromosomes. While targeting the centromeric repeats can eliminate the entire chromosome or may also produce isochromosomes. **(B)** The minichromosomes can be used as a shuttle system to stack multiple genes. **(C)** The isochromosomes produced by targeting DNA repeats present in centromere can generate new genetic variants. **(D)** The CRISPR/Cas9 targeting satellite DNA repeats can facilitate the faster introgression of target traits avoiding the requirement of long time period for backcrossing. **(E)** TnpBs can provide a miniature alternative to CRISPR/Cas system for chromosomal engineering.

The study by Chen et al. (2026) demonstrates that targeting satellite repeats by CRISPR/Cas9 results in modifications at the chromosomal level which include the truncation of terminal ends, loss of complete chromosome and formation of new chromosomes. Authors have concisely expressed this chromosomal engineering as “shrink, remove and modify”, which result from the large number of double-stranded cuts generated when Cas9 targets repetitive DNA sequences in the wheat genome (Figure 1A). The selection of the satellite DNA as target for CRISPR/Cas9 in this study serves to strengthen the concept of evolution of satellite DNA from a genetic ballast into a functional array which could be a valuable target for modern plant breeding tools. This study highlights that the selective targeting of large DNA repeat arrays results in either reduction in copy number or complete deletion of a particular repeat array which bring about chromosome-level modifications in the plant genome. A key strength of this work is that it demonstrates that both centromeric and non-centromeric satellite DNA repeats, although being located in heterochromatic region which typically limits the CRISPR/Cas machinery access to the single copy target gene and lowers the editing efficiency, can serve as a suitable target for CRISPR/Cas9-mediated chromosomal engineering. The high copy number of target sequences.i.e., DNA repeats, overcomes this limitation and generates significant DSB at multiple positions which overwhelm the cell’s repair machinery resulting in magnified genomic changes.

## Future implications and limitations

This remarkable achievement offers transformative potential for crop improvement and opens unprecedented opportunities to accelerate plant breeding. The potential of this strategy can be employed to generate minichromosomes providing a significant advantage over the previous top-down approaches for its development. The Agrobacterium-mediated transfer of telomere to induce chromosomal truncation results in insertion at random sites, which does not provide a control to target a particular chromosome and also leads to random downsizing of chromosomes (Yu et al., 2007). However, CRISPR/Cas9-based targeting of specific satellite repeats enables to target a particular chromosome at specific location resulting in the controlled terminal truncation. These minichromosomes can serve as a shuttle system to stack genes targeting multiple traits or polygenic traits and can be transferred to the next-generation as an all-gene set, extending the outcome beyond what traditional breeding methods can offer (Figure 1B). Also important to highlight, these chromosomal changes can refine existing cultivars and generate new genetic variants, paving the way for precision breeding and developing high-yielding climate resilient germplasm (Figure 1C). Moreover, the shrinkage or removal of chromosomes can prove to be a quicker approach to introgress valuable traits from wild relatives into wheat in comparison to the traditional breeding approaches which need several years of backcrossing to tackle the linkage drag (Figure 1D). Interestingly, the use of viral vectors in this strategy overcomes laborious and genotype-dependent traditional lengthy transformation procedures for different targets and can also be employed in recalcitrant plant species.

Despite the significant potential of this chromosomal engineering strategy, there are several challenges which must be addressed in order to ensure its application in crop breeding programs. The preliminary requirement of Cas9 transgenic lines in this BSMV-mediated chromosomal engineering experiment remained a technological bottleneck. In addition to BSMV, many other widely used viral vectors. e.g., CLCrV, TRV and PEBV reported as cargo carrier in genome editing experiments also cannot carry Cas9 protein due to their limited cargo size capacity (Lei et al., 2021; Ali et al., 2018; Oh and Kim, 2021). Thus, the use of VIGE strategies.e.g., fusion of sgRNA and Cas protein with mobile elements being delivered by viral vectors and use of viral vectors having large cargo capacity, can help to bypass the labour-intesive and time-consuming process of generating Cas9 transgenic lines providing a quicker approach to achieve genome editing (Ellison et al., 2020; Qiao et al., 2025). In addition to it, compact genome editors like Cpf1 and TnpBs, which can be easily delivered by viral vectors may also be deployed to create these chromosomal alterations providing a miniature alternative to Cas9 (Figure 1E) (Karvelis et al., 2021; Wu et al., 2021). The exploration of large repertoire of available viral vectors for genome editing and other DNA targeting Cas variants can help to expand the existing toolbox for chromosomal engineering. Besides using viral vectors, other carriers like nanoparticles and ribonucleoprotein complexes can also be exploited as suitable delivery methods. An important point of consideration is that this study does not provide precise control over the type of modifications being introduced. The detection of duplication events along with terminal truncation and generation of isochromosomes along with elimination of entire chromosome suggests that the use of CRISPR/Cas9 for chromosomal engineering must be carefully exploited to get the targeted and intended outcomes in plant genome. The current study has explored the use of CRISPR/Cas9 system for chromosomal engineering in wheat only. Thus, the same strategy must be investigated in other commercially important crops to increase its applicability in modern plant breeding aimed at developing climate resilient crops and achieving global food security. In addition, optimization to ensure complete removal of target repeats and integration of AI-assisted models for identification of optimal target satellite repeats, designing of guide-RNAs and prediction of resulting chromosomal modifications in plant genome could help to efficiently streamline this strategy for chromosomal engineering in complex plant genomes. This study by Chen and colleagues has provided a roadmap to utilize CRISPR/Cas9 for large-scale chromosomal modifications in polyploid crops strengthening the transformative potential of CRISPR/Cas systems for chromosomal engineering. However, future studies addressing these challenges may help to fully leverage the potential of this strategy in crop-breeding programs. In conclusion, this study by Chen et al. is just the beginning of a new genome editing era, in which genome editing for trait improvement in different crops will be addressed in a broader scope, targeting entire chromosomes from big and complex genomes rather than just focusing on a single gene.
